# Influence of Loading Rate and Temperature on the Energy Absorption of 3D-Printed Polymeric Origami Tubes under Quasi-Static Loading

**DOI:** 10.3390/polym14183859

**Published:** 2022-09-15

**Authors:** Xiubin Zuo, Chengjie Guo, Weidong Chen, Yixiao Wang, Jian Zhao, Huanlin Lv

**Affiliations:** 1 School of Textile and Material Engineering, Dalian Polytechnic University, Dalian 116034, China; 2 State Key Laboratory of Structural Analysis of Industrial Equipment, Dalian University of Technology, Dalian 116023, China; 3 School of Information Science and Engineering, Dalian Polytechnic University, Dalian 116034, China

**Keywords:** polylactic acid, 3D printing, energy absorption, finite element simulation, loading rate, temperature

## Abstract

Owing to deformation in the form of the diamond mode with high-energy absorption capacity, origami thin-walled tubes have attracted considerable attention in recent years. Stamping and welding are mainly employed to produce different types of origami thin-walled tubes. The processing defects and geometric asymmetry may be caused by the manufacturing process, which changes the collapsed mode and decreases the energy-absorbing capacity. In this study, fused filament fabrication (FFF) 3D printing is used to fabricate the origami-ending tube (OET) by integrated formation. Experiments and numerical simulations were conducted to study the influence of loading rate and temperature on the energy absorption of polymeric origami tubes under quasi-static loading. The experiments showed that different constitutive models are needed to capture the complex true stress–strain behavior of 3D printing polylactic acid (PLA) material at different temperatures. The damage model is established and then applied to the numerical simulations, which could predict the collapsed mode and the damage behavior of the OET tubes under different loading rates at 30 °C, 40 °C, and 50 °C. Based on the experiments and the validated numerical model, the influence of loading rate and temperature on the crashworthiness performance of the OET tubes is analyzed.

## 1. Introduction

A variety of thin-walled structures have been extensively used as energy absorbers [1,2] in various transportation vehicles to irreversibly convert kinetic energy into plastic deformation [3,4], friction [5,6], and material failure during impact accidents [7,8]. Owing to the deformation in predesigned buckling modes with lower initial peak force and higher specific energy absorption, the origami thin-walled tubes aroused much attention in this field [9,10]; however, the introduction of origami increases the difficulty of thin-walled tube fabrication. It has been shown that the origami thin-walled tubes are sensitive to manufacturing defects, especially for the non-integrated origami tubes [11,12]. In this investigation, a 3D printing technique is used to fabricate the origami-ending tubes.

The polymer can undergo large plastic deformations that make it particularly suited to applications where impact resistance is highly needed [13,14]; however, the polymeric structures have viscoelasticity and the polymer matrix is significantly sensitive to loading rate and temperature, which makes its crashworthiness more complicated under axial compression. Many studies [15,16,17,18] have been conducted on the loading rate and temperature effect on the mechanical properties of the polymeric structures and the polymer matrix composites. To the author’s knowledge, very few studies have investigated the influence of loading rate and temperature on the energy absorption of polymeric origami tubes under quasi-static loading.

In the last few years, theoretical prediction [19,20,21], experimental analysis [22,23,24], and numerical study [25,26,27] have been the three main research methods to investigate the crashworthiness of thin-walled structures under different loading conditions. Among them, the expression for the mean crushing force can be derived and modified by theoretical analysis; however, it is difficult to analyze the deformation details of the collapse mode of thin-walled structures through theoretical study. The experiments are the most direct investigated methods of thin-walled tubes, but have the disadvantages of being time-consuming and labor-intensive. Compared with the theoretical prediction and the experimental analysis, the numerical simulation method can conduct an in-depth analysis of the collapse mode and the main deformation mechanism of the energy-absorbing box, and can save labor and reduce costs.

In this paper, a polymer origami-ending tube (OET) is manufactured by fused filament fabrication (FFF) 3D printing technology [28,29]. Experimental tests and numerical simulation methods are introduced and implemented to evaluate the crashworthiness of OETs at different temperatures and loading rates. It is worth mentioning that 3D printing technology is used to ensure the integrated formation of these polymer origami tubes. The constitutive damage model is used to accurately characterize the stress–strain relationship of PLA and applied to numerical simulations to predict the collapse mode and damage behavior of OET tubes at different temperatures and loading rates. User-defined material subroutines for ABAQUS/Explicit have been developed for damage models. The layout of this paper is as follows. Section 2 gives the geometry of OET. The materials and methods are presented in Section 3. Section 4 uses the nonlinear finite element code ABAQUS to simulate the experimental tests. The results and discussions are shown in Section 5. Finally, conclusions are provided in Section 6.

## 2. Geometry

**Figure 1:**
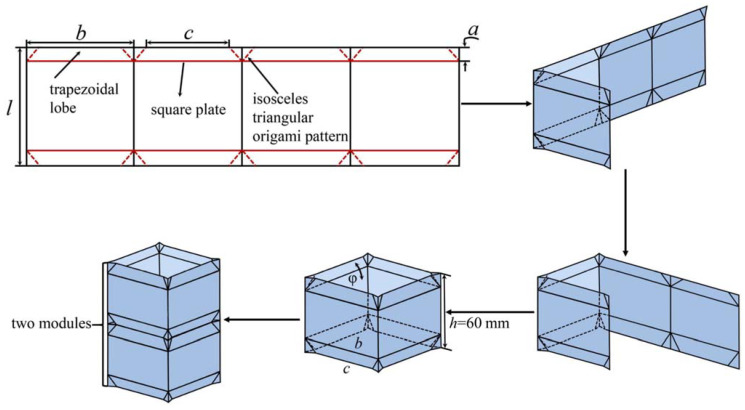
The traditional production process of OET.

Figure 1 shows that the OET is assembled by stacking one or several modules axially and is made by applying an isosceles triangular origami pattern (TOP) to the top and bottom ends. Mountain and valley creases are represented by the solid lines and the dashed lines, respectively. A typical module is built by folding an already prepared plate along the crease and then connecting two opposing free edges. The *l*, *b*, *c*, and *a* represent the tube length and width as well as the origami width and height, respectively. In this paper, the specific parameters of the model are *b* = 60 mm, *c* = 50 mm, *a* = 10 mm, and the height of a unit is *h* = 60 mm. In addition, the dihedral angle *φ* is the angle between the trapezoidal lobe and the square plate, which is the primary feature that distinguishes OET and conventional square tube (CST). Obviously, when the dihedral angle *φ* increases to 180°, OET will degenerate into CST. The detailed relationship of all geometric parameters can be found in Reference [30].

## 3. Materials and Methods

### 3.1. Materials

In this study, a new type of energy-absorbing box OET is researched, and the material of OET is mainly PLA. The material is derived from commercial material polylactic acid (PLA/1.75 mm) filament, produced by Shenzhen Creality 3D Technology Co., Ltd. (Shenzhen, China), and its density is 1.25 g/cm^3^. Models are formed by the FFF 3D printer CR-10S ( Shenzhen Creality 3D Technology Co., Ltd., Shenzhen, China). When using FFF technology to print PLA models, the setting of process parameters is very important. In this paper, the thickness of the printing layer is 0.2 mm, and the nozzle temperature, hot bed temperature, and printing speed are respectively 210 °C, 60 °C, and 50 mm/s. Since the layer-by-layer manufacturing process, the printing orientation has a significant effect on the mechanical properties. Taking into account the anisotropic properties of FFF 3D printing, all samples are printed along with the landscape orientation.

### 3.2. Tensile Test

To study the performance of 3D-printed polymer energy-absorbing boxes, the PLA material is first characterized. Dumbbell specimens are printed according to ASTM-D412, the quasi-static uniaxial tensile tests of the PLA material are carried out in a precision electronic universal testing machine AGS-X (Shimadzu Instruments Co., Ltd., Suzhou, Jiangsu, China), and the mechanical properties such as Young’s modulus and Poisson’s ratio of the material are obtained. The standard specimens are manufactured by 3D printing fused deposition, and its manufacturing process parameters and printing direction are the same as the OET model. The specimens are subjected to uniaxial tensile tests at different rates by using a precision electronic universal testing machine, and the stretching rates are 1, 5, and 10 mm/min, respectively. Experiments were performed in a temperature environmental chamber with heaters and circulating fans to characterize the mechanical properties of the printed materials at three different temperatures: 30 °C, 40 °C, and 50 °C. Before the specimens are stretched, it is held for 3 min to ensure the uniform temperature of the specimen. The specimen deformation is recorded using an electronic extensometer during the tensile experiments.

### 3.3. Quasi-Static Compression Test for OET

Quasi-static uniaxial compression testing of OET is performed using an AGS-X with a temperature-controlled chamber, and the OET is placed in the middle of two circular loading plates with one end fixed and the other loaded. In the test, displacement control loading is adopted, and the total loading distance is 65 mm. To evaluate the effect of temperature and loading rate on the energy absorbed by the model, the whole experiment is implemented at three different temperatures. The OET is preheated in the test environment before the experiment, and compression tests at three rates are carried out at each temperature. For each compression test, at least five trials in the same environment are performed for statistical significance. Displacement and compression force data are recorded by a machine data acquisition system, and video is used to document the compression process.

To quantify and evaluate the mechanical properties and energy absorption capacity of the specimens under axial compressive loading, five key indices are used in this study, including initial peak force ($F_{\max}$), average force ($F_{m}$), total energy absorption specific ($E_{\mathrm{total}}$), energy absorption (*SEA*), and crushing force efficiency (*CFE*). The expressions of these indicators are calculated as follows:

(1) $$E_{\mathrm{total}}=\int_{0}^{\delta }F(s)\mathrm{ds}$$

(2) $$\mathrm{SEA}=\frac{E_{\mathrm{total}}}{m}=\frac{\int_{0}^{\delta }F(s)\mathrm{ds}}{m}$$

(3) $$F_{m}=\frac{E_{\mathrm{total}}}{\delta }=\frac{\int_{0}^{\delta }F(s)\mathrm{ds}}{\delta }$$

(4) $$\mathrm{CFE}=\frac{F_{\max}}{F_{m}}$$

where δ is the final compression distance, *m* is the total mass of the tube. The specific meaning of these indicators can be found in [31,32].

## 4. Numerical Simulation

### 4.1. The Damage Model

Due to the brittleness of PLA at 30 °C and 40 °C, the material suffered damage in quasi-static compression. To describe the mechanical behavior of PLA under quasi-static loading, a damage model with the damage variable *D* is considered. Based on the concept of effective stress, the damage model is summarized as the following equations in uniaxial loading [33]:

(5) σ=(1−D)Eε

σ and ε are the stress and strain, respectively, *E* is the initial Young’s modulus, and *D* is the damage variable and is given by [33,34]:

(6) $$D=\left\{\begin{array}{c} \;0,\;(0\;\leq \;\varepsilon \;\leq \varepsilon_{f})\\ 1-\frac{\varepsilon_{f}\;(1-{\;C}_{1})}{\varepsilon }-\frac{A_{1}}{\exp\;\left[B_{1\;}(\varepsilon -{\;\varepsilon }_{f})\right]}\;,\;\;\;\;\;\;\;\;\;\;{\;(\varepsilon \;>\;\varepsilon }_{f})\; \end{array}\right.$$

$A_{1}$, $B_{1}$, and $C_{1}$ denotes material constants, and $\varepsilon_{f}$ denote the initial damage strain.

Among them, the maximum equivalent plastic strain criterion is used. In the finite element simulation, when the equivalent plastic strain of a material element exceeds the maximum equivalent plastic strain, the element is deleted.

### 4.2. Finite Element Modeling (FEM)

**Figure 2:**
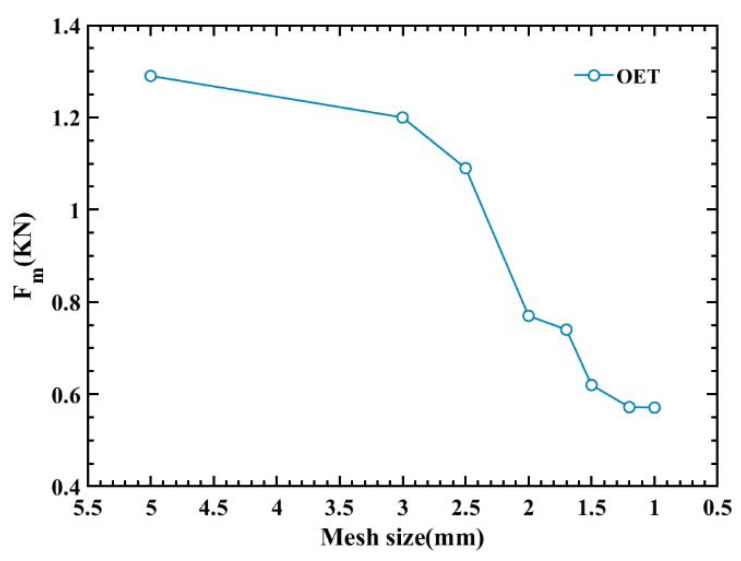
Mesh sensitivity test of the OET.

**Figure 3:**
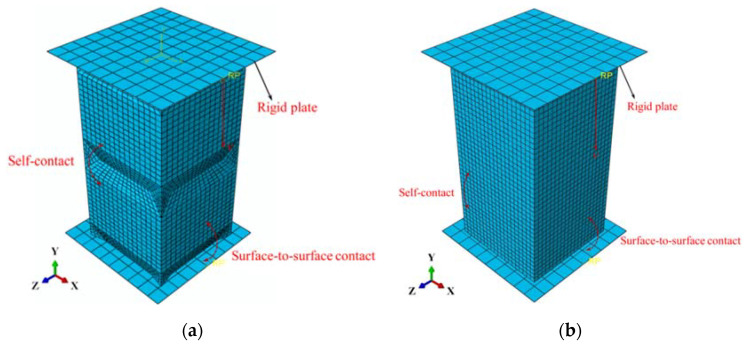
(**a**) Finite element model of the OET; (**b**) finite element model of the CST.

Finite Element (FE) models of the axial crushing of the OET are constructed by commercial Finite Element Software ABAQUS (6.14, Providence, RI, USA); within this, the model is solved in solver ABAQUS/Explicit. The tube wall is meshed using reduced-integration four-node (S4R) shell elements. On the one hand, the simulation results are closely related to the grid density, and enough mesh density is required to guarantee the accuracy of the result; on the other hand, extremely small mesh sizes can also lead to long simulation times. To determine the optimal mesh size for numerical simulations, a convergence test of the mesh density is conducted. As shown in Figure 2, the convergence tests of eight different mesh sizes are through, the accuracy and calculation time are considered, and an average grid size of 1 mm is chosen. To apply an axial compressive load to the OET, two rigid plates are attached to two ends of the OET in the axial direction. Surface-to-surface contact is used between the OET and the rigid plates; in addition, the tube wall uses self-contact, a friction coefficient of 0.3 is applied to the whole model. During axial compression, the motion of the bottom rigid plate is fully constrained in all directions, and the upper rigid plate is displaced 65 mm to the lower plate in the axial direction, as shown in Figure 3. In order to minimize the dynamic and hourglass effects, the ratios of both kinetic energy to internal energy as well as artificial energy to internal energy are less than 5%. Furthermore, a smooth amplitude, which is defined to build in ABAQUS, is applied to control the loading rate of the top plate to ensure that the quasi-static results are acquired. The material property of OET in this study is obtained by conducting axial tensile tests of the test sample of PLA, and input into ABAQUS by using user-defined material subroutine VUMAT, the values of main mechanical properties are summarized in Table 1.

## 5. Result and Discussion

### 5.1. Experimental and Simulation Analysis

To validate the finite element method, under a compression rate of 5 mm/min at 40 °C, the simulation results of CST under quasi-static compression are compared with the experimental data, and the detailed data are summarized in Table 2. As can be seen from Table 2, the relative errors of peak and mean forces between the numerical simulation as well as the experimental are approximately 7.59% and 2.01%, respectively.

**Figure 4:**
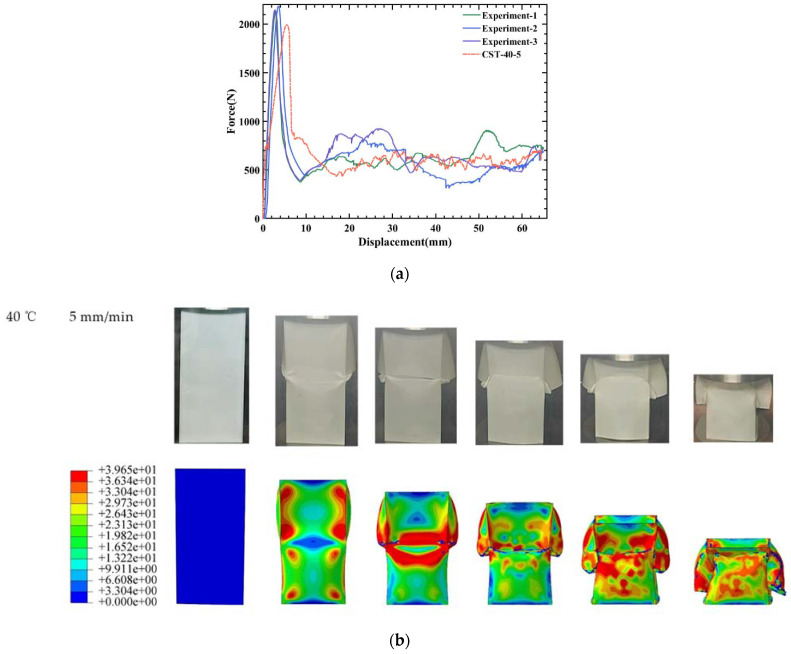
Validation of the finite element model: (**a**) force–displacement curves of experimental and finite element results; (**b**) comparison of deformation modes in the experiment and simulation.

Figure 4 shows the comparison of the overall process of the CST experiment and numerical simulation. It can be seen from Figure 4a that the overall trends of the force–displacement curve between the numerical simulation and the experiment are quite similar. For the deformation mode of CST, as shown in Figure 4b, in the experimental test, local buckling occurs in the middle part, then a brittle crack occurs at the buckling of the tube wall; as the compression progresses, the cracks expand. The deformation mode obtained by the numerical simulation is consistent with the experiment; however, there are still some mismatches between experiments and numerical simulations. This is mainly because the bottom of the experimental model is free in contact with the bottom surface of the universal testing machine, while in the finite element simulation process, the bottom of the model and the lower rigid plate are fixed constraints. Additionally, these discrepancies may be due to the deletion of the failed elements in the simulation, while the failed part still exists in the experiment. Overall, the evaluation parameters are similar between experiments and numerical simulations, and the finite element method can accurately predict the mechanical properties and deformation modes of the tubes.

### 5.2. The Relationship between OET Compression Performance and Rate

**Figure 5:**
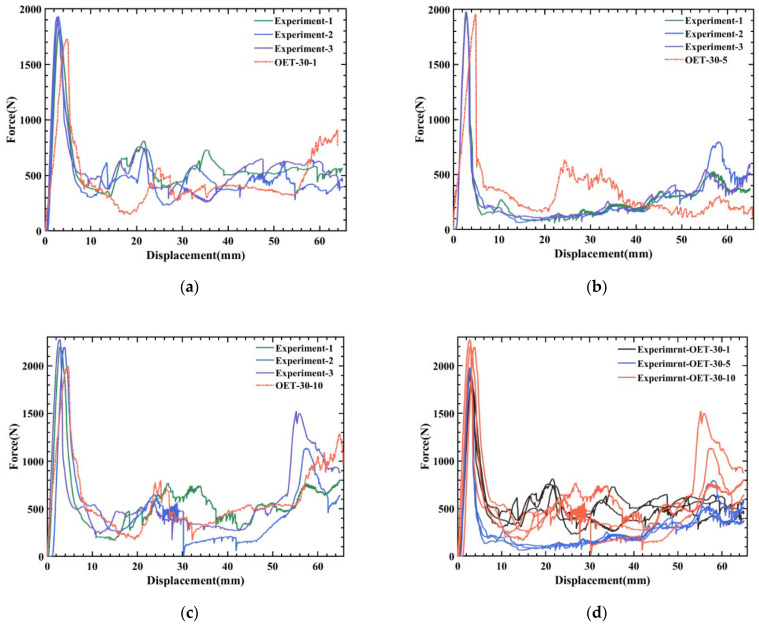
Force–displacement curves of OET loading rate at 30 °C: (**a**–**c**) are the comparison between experiment and simulation when the compression rate at 1 mm/min, 5 mm/min, and 10 mm/min, respectively. (**d**) Comparison of different loading rates.

**Figure 6:**
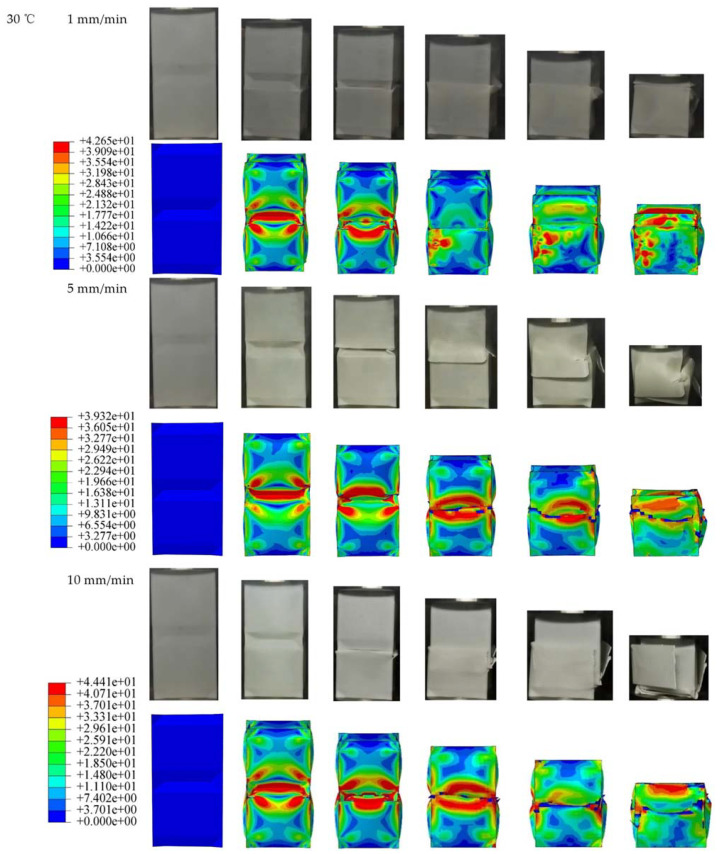
Experiment and simulation of deformation process of OET.

To research the relationship between the compression performance of OET and rate, the compression at different rates under the same temperature is analyzed. Figure 5 plots the experimental and simulated force–displacement curves for three different loading rates at 30 °C. The corresponding OET deformation process and detailed data are shown in Figure 6 and Table 3, respectively. The maximum error in Table 3 is 16.67%, and it can be seen the numerical value of the calculated curves and deformation modes are in good agreement with the experimental results within the allowable error range.

**Figure 7:**
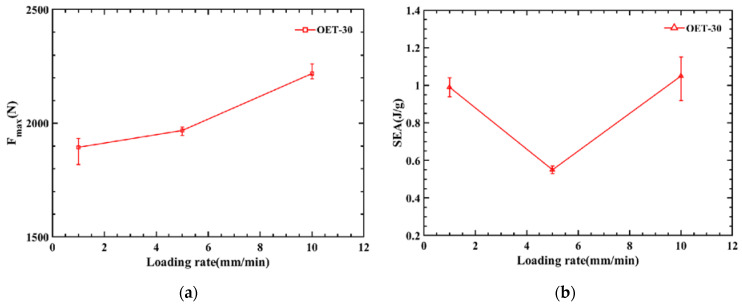

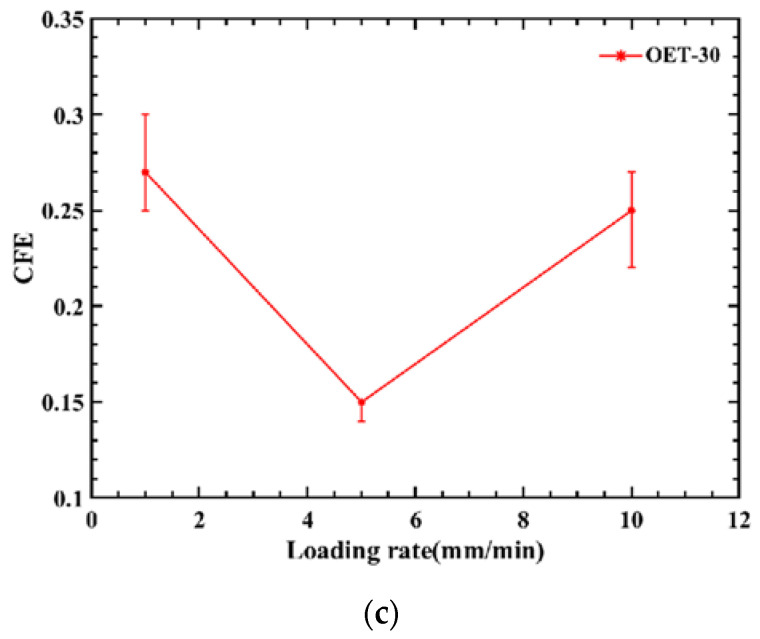
Dynamic performance of OET at different rates: (**a**) $F_{\max}$, (**b**) *SEA*, and (**c**) *CFE*.

To analyze the dynamic changes of various properties during the compression process, Table 4 and Figure 7 list the various performance indicators and their changing trends in the OET experiment in detail. Figure 7a shows the relationship between compression rate and the maximum peak force of the force–displacement curve; it is concluded that the maximum peak force of the force–displacement curve increases with the compression rate. Due to the increased compression rate, the strength and stiffness of the material rise. Figure 7b,c show the trend graphs of *SEA* and *CFE*, respectively. Significantly that both have a minimum value at a rate of 5 mm/min, and this phenomenon is explained in conjunction with the compression deformation process of the experiment and simulation in Figure 6.

All OETs are observed to deform from the origami end, as shown in Figure 5d; when the curve reached the maximum peak, the displacement is 3–5 mm, and according to the Mises stress cloud map, the stress is concentrated at the junction of the two modules. As the compressive displacement increases, brittle cracking occurs in the middle region of the tube, at which time the load drops sharply. When the displacement continued to increase, two different deformation features appeared, namely nesting (1 mm/min and 10 mm/min) and tilting (5 mm/min).

The nesting is characterized by local buckling in the middle of the OET with small cracks extending along the circumference of the shell. When cracks grow in the middle of the tube, they divide the tube into two separate parts. As compression progresses, the upper module slides and is then inserted into the inner hollow of the lower module. Under a rate of 10 mm/min, the stiffness of PLA is large, which leads to severe embrittlement of the lower module, and a compaction phenomenon occurs in the final stage of compression, and the load increases, thereby improving the overall *SEA* and *CFE*. At a rate of 5 mm/min, the two modules first buckled at the connection corner. As the displacement increases, the upper module cracks at the connection corners with higher stress, and the OET tilts.

In conclusion, when the compression rate is 1 mm/min, the peak force is the lowest, the *SEA* is relatively high, and the deformation mode of OET is stable. According to the specific values of various properties, the energy absorption effect is the best.

### 5.3. The Effect of Temperature on OET

**Figure 8:**
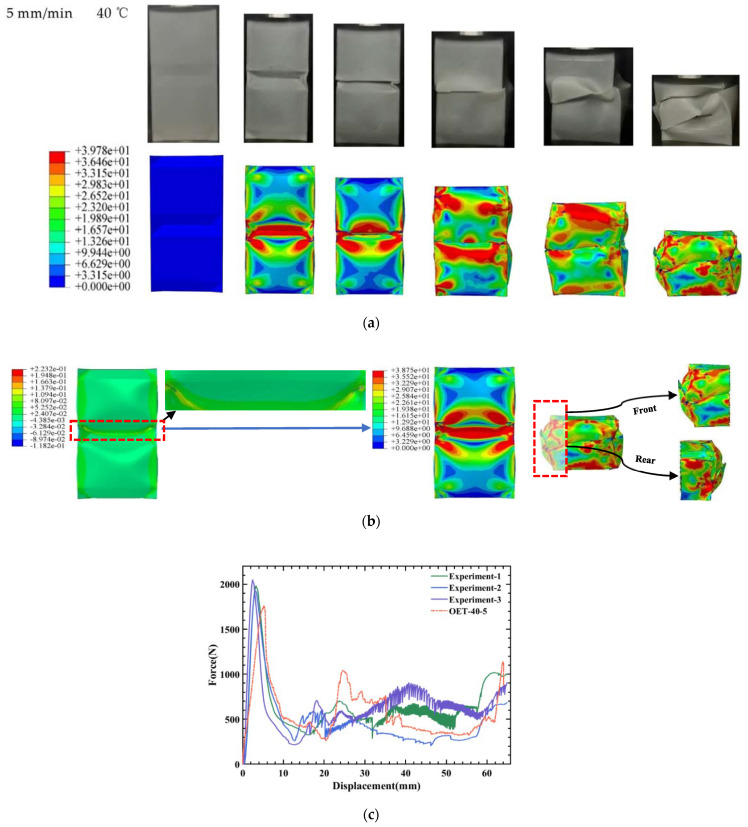
The compression rate at 5 mm/min: (**a**) Comparison of deformation modes between OET axial compression experiment and numerical simulation at 40 °C; (**b**) strain and stress cloud maps of OET at 40 °C; (**c**) comparison of the experimental and simulated force–displacement curves of OET at 40 °C.

To understand the effect of temperature on the deformation mode and the energy absorption behavior of OET. The quasi-static compression process at different temperatures is studied at a compression rate of 5 mm/min. The force–displacement curves and deformation modes at 30 °C are shown in Section 5.2. Figure 8a alone shows the deformation modes during the experiment and numerical simulation of OET at 40 °C. It can be seen due to the local stress concentration, the buckling of the tube occurs first at the connection of the two modules. In this stage, the local tube wall buckling is unstable, brittle fracture damage occurs, and symmetrical cracks are generated, as shown in Figure 8b. With the ongoing compression, this phenomenon propagates to two modules. Two modules experience extrusion deformation as these cracks expand. Figure 8b shows the details of the later stage of extrusion deformation. To be specific, the rear of the OET causes a phenomenon of two modules nesting while the front is not nested. This results in tilting and misalignment of the OET.

The corresponding force–displacement curves are shown in Figure 8c. Obviously, numerical simulations and experiments show almost the same response trends. To be specific, the deformation process can be divided into two stages: before and after the damage stage. In the first stage, the top plate is in contact with the OET and the force–displacement curve increases rapidly up until the peak force $F_{\max}$ is reached. Subsequently, the force begins to decrease to the valley due to the brittle fracture of the OET. Finally, the curve is in a relatively flat stage. During the whole deformation process, $\Delta F_{\max}$ and Δ*SEA* of experimental as well as numerical simulations are 11.10% and 4.46%, respectively; the detailed data are shown in Table 5. Therefore, the numerical simulation results agree well with the experimental results.

**Figure 9:**
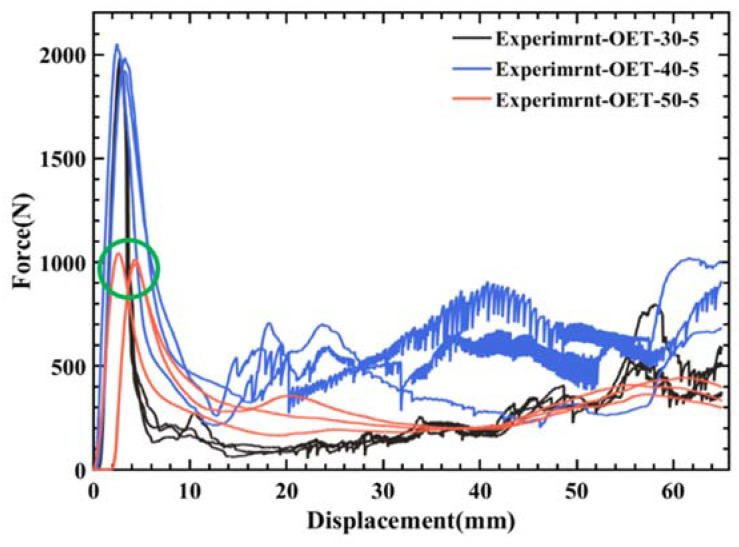
The compression rate at 5 mm/min: force–displacement curves of OET axial compression experiment at 30 °C, 40 °C, and 50 °C.

**Figure 10:**
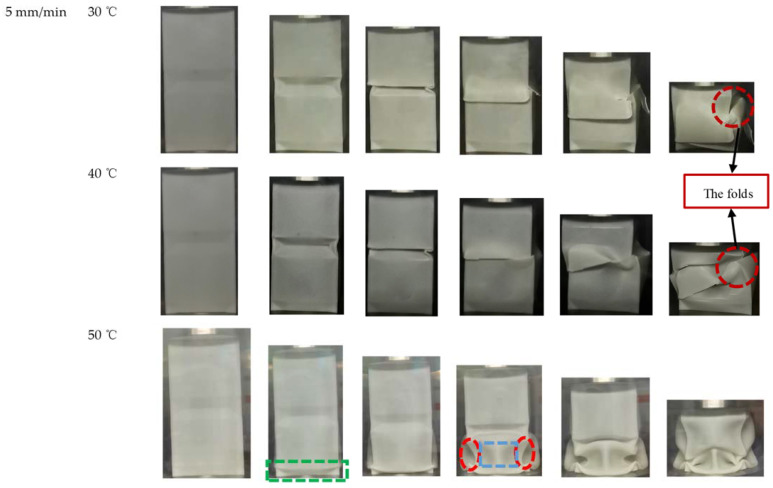
The compression rate at 5 mm/min: deformation modes of OET axial compression experiment at 30 °C, 40 °C, and 50 °C.

At 50 °C, due to the complex constitutive relation of PLA material at high temperature, there is still no reliable method to realize it in finite element software. Therefore, the influence of 50 °C on the deformation mode of OET has been mainly studied experimentally. Table 6 shows the energy absorption index of uniaxial quasi-static compression experiments and numerical simulations of OET at a compression rate of 5 mm/min and a temperature of 50 °C. The specific force–displacement curves and deformation mode are shown in Figure 9 and Figure 10. It can be seen that the OET is deformed from the bottom origami end (marked by the green rectangle in Figure 10). At the same time, the force–displacement curve reaches the peak force (marked by the green circle in Figure 9). With the ongoing compression, the force decreases to the valley under the influence of the end origami initiators. The blue rectangular region in Figure 10 moves outward, and the red ellipse region moves inward, forming two traveling plastic hinge lines on both sides of the corner area of the specimen. Subsequently, the corner area at the connection of the two modules begins to deform, and the above phenomenon propagates from one module to the other.

**Figure 11:**
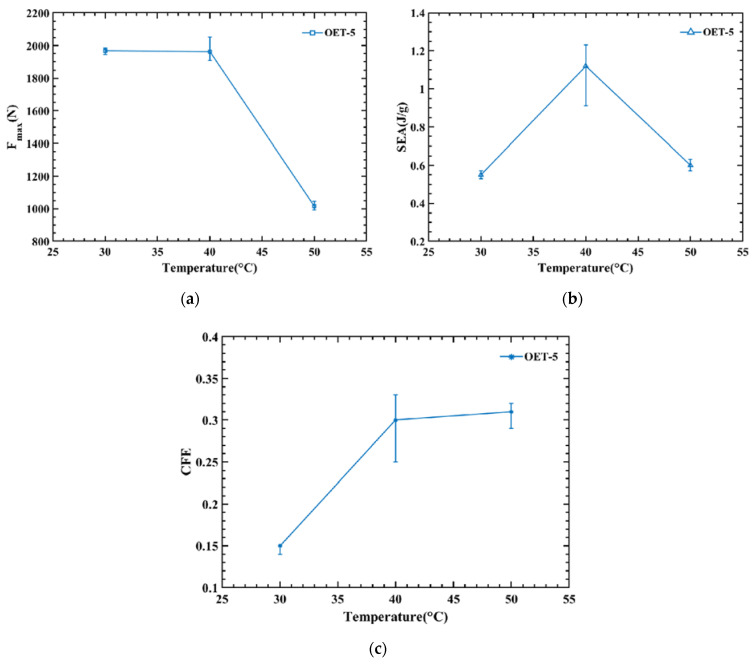
The effect of temperature on three Energy absorption indicators: (**a**) $F_{\max}$, (**b**) *SEA*, and (**c**) *CFE*.

Based on the above experimental analysis, the energy absorption indices of OET at three temperatures are shown in Table 7 and Figure 11. Figure 11a shows the relationship between temperature and peak load $F_{\max}$, it can be seen the peak force decreases with increasing temperature. The reason for this is that the increase in temperature reduces the strength and stiffness of PLA. Except for the peak load, the *SEA* and *CFE* are also affected by temperature. As shown in Figure 11b,c, *SEA* shows a maximum at 40 °C, while *CFE* rises with increasing temperature. As can be seen from Figure 10, the OET developed cracks at both 30 °C and 40 °C, and the cracks appeared in the middle of the tube, which forced the tube to produce folds in the middle of the tube. This deformation feature impairs the energy absorption capacity. The cracks are significantly larger at 30 °C, resulting in lower *SEA* and *CFE*. While at 50 °C, the high temperature reduces the stiffness of PLA, which in turn leads to lower *SEA*, and its higher *CFE* is due to a significant reduction in peak load rather than an increase in average load. In summary, the temperature rise will make the OET have better energy absorption performance.

### 5.4. Comparison between Different Types of Tubes

**Figure 12:**
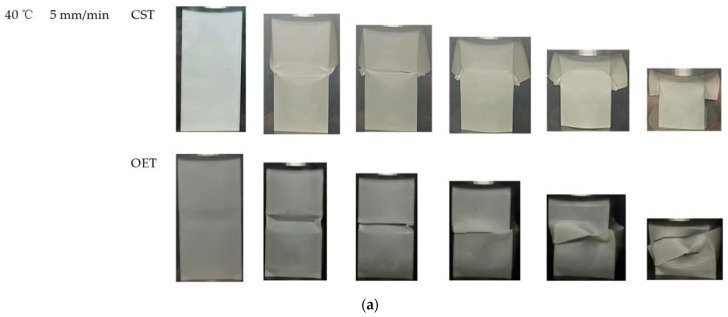

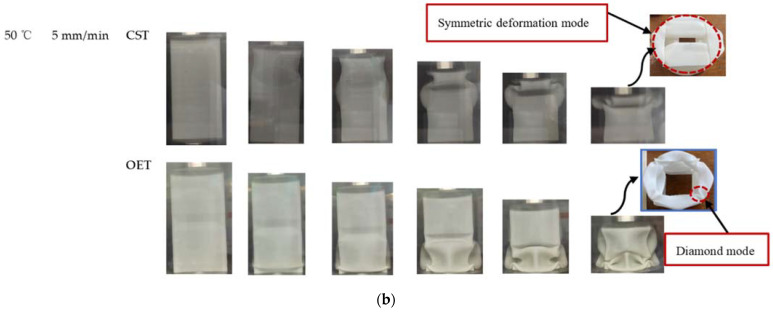
The compression rate at 5 mm/min: (**a**) Deformation mode of OET and CST at 40 °C; (**b**) deformation mode of OET and CST at 50 °C.

**Figure 13:**
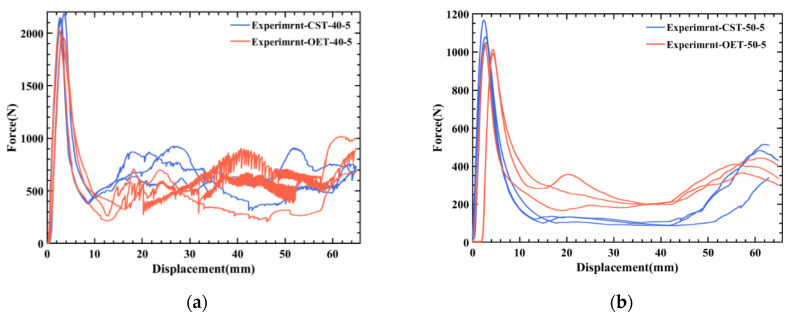
The compression rate at 5 mm/min: comparison of force–displacement curves of CST and OET: (**a**) 40 °C; (**b**) 50 °C.

In this section, Table 8 shows that experimental results of CST at 50 °C. The effect of temperature on deformation mode and energy absorption is exhibited by comparing CST and OET. Compared with CST, $F_{\max}$ of OET is reduced by 9.05% and 7.85%, respectively, when the temperature increases from 40 °C to 50 °C, as shown in Table 9. The main reason is that the trapezoidal lobes near the end of OET are inclined, which results in an obvious reduction in axial stiffness compared to CST, as illustrated in Figure 12a. It also can be seen from Figure 13 that curves of OET are gradually higher than that of CST as the temperature increases, which means that the OET will absorb more energy. In comparison to CST, the *SEA* value of OET increased from 90.33% for CST to 122.45% for CST, as the temperature rises from 40 °C to 50 °C, as summarized in Table 9. This could be explained by the effect of temperature on deformation modes. Evidently, brittle cracking occurs in both OET and CST when the temperature is 40 °C, which results in the isosceles triangle origami pattern introduced to improve the energy absorption effect of CST will not be able to play its role or even become a defect, which makes the energy absorption effect of the OET slightly worse than that of CST. On the contrary, at a temperature of 50 °C, neither OET nor CST crack due to the sensitivity of PLA to temperature. In this case, for CST, only one moving plastic hinge line is formed at each corner, and symmetric deformation mode is triggered in CST. In contrast, the deformation process of OET is guided by an isosceles triangle origami pattern; two moving plastic hinges are formed at each corner; this new deformation mode is called complete diamond mode, as presented in Figure 12b. According to the super-folding element theory [35], more moving plastic hinges lead to more plastic deformation and more energy absorption. Furthermore, the *CFE* of OET is markedly improved compared to the *CFE* of CST due to the rise in temperature. Thus, it can be concluded that when plastic deformation occurs, OET can absorb more energy, and the energy absorption capacity is better than CST.

## 6. Conclusions

In this paper, we investigate the response process of the PLA 3D-printed OET model under quasi-static compressive loading at different loading rates and temperatures, and the following conclusions are shown:

(1) To accurately predict the mechanical behavior of the model, the simulation results are compared with the experimental data, and the consequence shows that the simulation results are consistent with the experimental results;
(2) At the same temperature, due to the rate sensitivity of PLA material, when the rate is 1 mm/min, OET has the best energy absorption effect;
(3) Comparing the experimental results of the OET model at the same rate and different temperatures, it is determined that the *CFE* of OET rises with increasing temperature, which reflects the temperature sensitivity of PLA materials;
(4) When OET is without brittle cracking, its energy absorption capacity is better than that of CST. When the OET is brittle and cracked, its structural superiority is not reflected.

## Figures and Tables

**Table 1:** Experimental parameters of FFF 3D-printed PLA tensile specimens.

Temperature	Rate (mm/min)	Density	Elastic Modulus (MPa)	Poisson’s Ratio
30 °C	1	1250 kg/m^3^	1525	0.36
	5	1250 kg/m^3^	2341	0.36
	10	1250 kg/m^3^	2277	0.36
40 °C	1	1250 kg/m^3^	1440	0.36
	5	1250 kg/m^3^	1650	0.36
	10	1250 kg/m^3^	1746	0.36

**Table 2:** Comparison of simulation results with experimental results.

Temperature	Rate (mm/min)	Specimen	$F_{\max}\;(N)$	$E_{\mathrm{total}}\;(J)$	${\;F}_{m}\;(N)$	*SEA* (J/g)	$\Delta F_{\max}$ (%)	$\Delta F_{m}$ (%)	ΔSEA (%)
40 °C	5	Experiment-1	2131.63	43.48	668.92	1.26	-	-	-
		Experiment-2	2188.70	40.46	622.46	1.17	-	-	-
		Experiment-3	2148.93	44.80	689.23	1.29	-	-	-
		EA	2156.42	42.91	660.15	1.24	-	-	-
		CST-40-5	1992.64	43.79	673.69	1.27	7.59	2.01	2.36

**Table 3:** Data comparison between the experimental and simulation results of OET.

Temperature	Rate (mm/min)	Specimen	$F_{\max}\;(N)$	$E_{\mathrm{total}\;}\left(J\right)$	${\;F}_{m}\;(N)$	*SEA* (J/g)	*CFE*	$\Delta F_{\max}$ (%)	ΔSEA (%)	ΔCFE (%)
30 °C	1	Experiment-1	1817.85	35.47	545.69	1.04	0.30	-	-	
		Experiment-2	1932.66	31.85	490.00	0.94	0.25	-	-	
		Experiment-3	1931.26	33.87	521.08	0.10	0.27	-	-	
		EA	1893.92	33.73	518.92	0.99	0.27	-	-	
		OET-30-1	1728.51	29.72	457.23	0.87	0.26	8.73	12.12	3.70
	5	Experiment-1	1983.79	18.14	279.11	0.53	0.14	-	-	
		Experiment-2	1946.65	19.27	296.53	0.57	0.15	-	-	
		Experiment-3	1973.05	18.95	291.56	0.56	0.15	-	-	
		EA	1967.83	18.79	289.07	0.55	0.15	-	-	
		OET-30-5	1959.10	22.62	348.08	0.66	0.18	0.44	16.67	16.67
	10	Experiment-1	2200.28	37.04	569.85	1.09	0.26	-	-	
		Experiment-2	2194.31	31.34	482.15	0.92	0.22	-	-	
		Experiment-3	2260.71	39.26	604.00	1.15	0.27	-	-	
		EA	2218.43	35.88	552.00	1.05	0.25	-	-	
		OET-30-10	1995.38	39.24	603.70	1.15	0.30	10.05	9.52	16.67

**Table 4:** The specific data of each evaluation index of OET.

Temperature	Rate (mm/min)	Specimen	$F_{\max}\;(N)\;$	$F_{m}\;(N)\;$	*SEA* (J/g)	*CFE*
30 °C	1	OET-EA-30-1	1893.92	518.92	0.99	0.27
	5	OET-EA-30-5	1967.83	289.07	0.55	0.15
	10	OET-EA-30-10	2218.43	552.00	1.05	0.25

**Table 5:** The compression rate at 5 mm/min: the energy absorption index of quasi-static uniaxial compression experiment and numerical simulation of OET at 40 °C.

Rate (mm/min)	Temperature	Specimen	$F_{\max}\;(N)$	$E_{\mathrm{total}}\;(J)$	${\;F}_{m}\;(N)$	*SEA* (J/g)	$\Delta F_{\max}$ (%)	ΔSEA (%)
5	40 °C	Experiment-1	1906.96	41.49	638.30	1.22	-	-
		Experiment-2	1924.47	31.05	477.69	0.91	-	-
		Experiment-3	2052.02	41.78	642.77	1.23	-	-
		EA	1961.15	38.11	586.31	1.12	-	-
		OET-40-5	1743.38	39.68	610.46	1.17	11.10	4.46

**Table 6:** The compression rate at 5 mm/min: the energy absorption index of quasi-static uniaxial compression experiment and numerical simulation of OET at 50 °C.

Rate (mm/min)	Temperature	Specimen	$F_{\max}\;(N)$	$E_{\mathrm{total}}\;(J)$	${\;F}_{m}\;(N)$	*SEA* (J/g)
5	50 °C	Experiment-1	991.92	20.11	309.38	0.59
		Experiment-2	1014.01	21.33	238.15	0.63
		Experiment-3	1044.12	19.35	297.69	0.57
		EA	1016.68	20.26	311.69	0.60

**Table 7:** Energy absorption index of OET at different temperatures when the compression rate at 5 mm/min.

Rate (mm/min)	Temperature	Specimen	$F_{\max}\;(N)\;$	${\;F}_{m}\;(N)\;$	*SEA* (J/g)	*CFE*
5	30 °C	OET-EA-30-5	1967.83	289.07	0.55	0.15
	40 °C	OET-EA-40-5	1961.15	586.31	1.12	0.30
	50 °C	OET-EA-50-5	1016.68	311.69	0.60	0.31

**Table 8:** Experimental results of CST at 50 °C.

Temperature	Rate (mm/min)	Specimen	$F_{\max}\;(N)$	$E_{\mathrm{total}}\;(J)$	${\;F}_{m}\;(N)$	*SEA* (J/g)
50 °C	5	CST-Experiment-1	1080.84	14.63	225.08	0.42
		CST-Experiment-2	1168.10	20.75	319.23	0.60
		CST-Experiment-3	1051.08	15.73	242.00	0.45
		EA	1100.01	17.04	262.15	0.49

**Table 9:** Experimental results of OET and CST at 40 °C and 50 °C.

Rate (mm/min)	Temperature	Specimen	b (mm)	H (mm)	$F_{\max}$ (N)	${\;F}_{m}$ (N)	*SEA* (J/g)	*CFE*	$\Delta F_{\max}$ (%)	ΔSEA (%)	ΔCFE (%)
5	40 °C	CST-EA-40-5	60.00	120.00	2156.42	660.15	1.24	0.31	-	-	-
		OET-EA-40-5	60.00	120.00	1961.15	586.31	1.12	0.30	9.05	9.67	3.23
	50 °C	CST-EA-50-5	60.00	120.00	1100.01	262.15	0.49	0.24	-	-	-
		OET-EA-50-5	60.00	120.00	1016.68	311.69	0.60	0.31	7.58	22.45	29.17

## Data Availability

The raw/processed data required to reproduce these findings cannot be shared publically but is available upon request.
